# Physiochemical property space distribution among human metabolites, drugs and toxins

**DOI:** 10.1186/1471-2105-10-S15-S10

**Published:** 2009-12-03

**Authors:** Varun Khanna, Shoba Ranganathan

**Affiliations:** 1 Dept. of Chemistry and Biomolecular Sciences & ARC Centre of Excellence in Bioinformatics, Macquarie University, Sydney, Australia; 2 Dept. of Biochemistry, Yong Loo Lin School of Medicine, National University of Singapore, Singapore

## Abstract

**Background:**

The current approach to screen for drug-like molecules is to sieve for molecules with biochemical properties suitable for desirable pharmacokinetics and reduced toxicity, using predominantly biophysical properties of chemical compounds, based on empirical rules such as Lipinski's "rule of five" (Ro5). For over a decade, Ro5 has been applied to combinatorial compounds, drugs and ligands, in the search for suitable lead compounds. Unfortunately, till date, a clear distinction between drugs and non-drugs has not been achieved. The current trend is to seek out drugs which show metabolite-likeness. In identifying similar physicochemical characteristics, compounds have usually been clustered based on some characteristic, to reduce the search space presented by large molecular datasets. This paper examines the similarity of current drug molecules with human metabolites and toxins, using a range of computed molecular descriptors as well as the effect of comparison to clustered data compared to searches against complete datasets.

**Results:**

We have carried out statistical and substructure functional group analyses of three datasets, namely human metabolites, drugs and toxin molecules. The distributions of various molecular descriptors were investigated. Our analyses show that, although the three groups are distinct, present-day drugs are closer to toxin molecules than to metabolites. Furthermore, these distributions are quite similar for both clustered data as well as complete or unclustered datasets.

**Conclusion:**

The property space occupied by metabolites is dissimilar to that of drugs or toxin molecules, with current drugs showing greater similarity to toxins than to metabolites. Additionally, empirical rules like Ro5 can be refined to identify drugs or drug-like molecules that are clearly distinct from toxic compounds and more metabolite-like. The inclusion of human metabolites in this study provides a deeper insight into metabolite/drug/toxin-like properties and will also prove to be valuable in the prediction or optimization of small molecules as ligands for therapeutic applications.

## Background

To search for biologically active compounds, with favorable ADMET [1] (Absorption, Distribution, Metabolism, Excretion, and Toxicity) properties from the immense "chemical space" is a non-trivial task [2]. Drug-likeness has been dominated, in the past decade, by Lipinski's "Rule of Five" (Ro5) [3], which states that a compound is likely to be "non-drug-like" if it has more than five hydrogen bond donors, more than 10 hydrogen bond acceptors, molecular mass is greater than 500 and lipophilicity is above 5. The analysis carried out by Leeson and Davis [4] of the approved drugs released before 1983 (i.e. pre-Ro5 era) and the drugs released in between 1983 and 2002 clearly indicates the impact of Ro5 on drug discovery projects.

However, Lipinski's rule has many exceptions and in one of the studies [5] it was shown that using the above criteria, only 66% of approved drugs in the MDL Drug Data Report (MDDR) database, were classified as drug-like; whereas 75% of the theoretically non-drug-like compounds from the Available Chemical Directory (ACD) were in fact regarded as drug-like by Ro5. Moreover, Ro5 does not select metabolites because metabolite-likeness is a recent measure, since Ro5 was formulated a decade ago, with little knowledge on metabolites and pathways. Similar studies have spurred the quest for new approaches to classify drugs from non-drug molecules [6,7], and to characterize the properties of drug-like or lead-like compounds [8,9]. Subsequently, the "rule-of-three" (Ro3) [10] was proposed for fragment-based lead discovery. Ro3 states that successful hits possess an average Molecular weight <= 300, the number of hydrogen bond donors <= 3, the number of hydrogen bond acceptors <= 3 and Clog P <= 3. In addition, the number of rotatable bonds <= 3 and the polar surface area <= 60 are also useful in characterizing drug-like and non-drug-like molecules. In past few years, researchers have developed a range of indices, such as the natural product index [11], the metabolite index [12], peptide-likeness [13], lead-likeness [14-16], and drug-likeness [3], in an attempt to achieve a better classification between drugs and non-drugs. In conjunction with machine learning techniques, like Artificial Neural Networks (ANN) [6,7], Support Vector Machine (SVM) [17] and Hidden Markov Models (HMM), statistical [18] and substructure analyses have become widely accepted to characterize the properties of drug-like datasets and reduce the attrition rates in drug development.

### Drug-likeness in natural products and synthetic compounds

In this section, we present a summary of analysis reports primarily focused on identifying drug-likeness in natural products and synthetic organic compounds, derived from combinatorial functional group replacement. Henkel et al. [18] carried out statistical analysis to determine the properties and structural differences between natural products (NPs) and combinatorial molecules. In their analysis, NPs were derived from Chapman and Hall Dictionary of Natural products and the bioactive natural product database (BNPD) obtained from Szenzor Management Consulting Company. These were compared with synthetic compounds from the Available Chemical Directory (ACD) and Bayers database and representative bioactive molecules from drug databases. Stahura et al. [19] used Shannon entropy to analyze the differences between NPs obtained from the Dictionary of Natural Products and synthetic molecules obtained from ACD database. Feher and Schmidt [20] examined representative set of molecules from NPs obtained from four databases namely BioSPECS natural product database, ChemDiv natural product database, Interbioscreeen IBS2001N and HTS-NC database, drugs obtained from (Chapman and Hall Dictionary of Drugs) and combinatorial molecules obtained from (MayBridge HTS database, ChemBridge EXPRESS-Pick database ComGenex Collection, ChemDiv Collection and SPECS screening compound database). The authors concluded that the number of chiral centers, the number of rotatable bonds and the ratio of aromatic atoms to ring atoms are the most distinguishing features among the three classes of compounds. In their study, drugs occupied the property space between NPs and combinatorial compounds, consistent with drugs being obtained from NPs as well as combinatorial libraries. The first three principal components accounted for about 66% of the variance. Feher and Schmidt were thus the first to introduce the idea of NP-like filters. Lee and Schneider [21] utilized Self Organizing Maps (SOM) for the classification of drugs, non-drugs and NPs. Their study revealed several pharmacophoric patterns in common between NPs and drugs, suggesting the use of such patterns for exploring drug relevant pharmacophoric space.

### Metabolite-likeness as the criterion for lead discovery

With the growing knowledge of biochemical pathways and their cognate metabolites, Hattori et al. [22] analyzed the molecular diversity of KEGG (Kyoto Encyclopedia of Genes and Genomes) Ligand database which includes 9,383 chemical structures. Nobeli et al. [23] have produced an interesting classification of *Escherichia coli *metabolome according to fragment-based fingerprints and maximum common subgraphs. Gupta and Aires-de-Sousa [12] compared the structural coverage of the metabolite molecules from the KEGG database and purchasable molecules from the ZINC library, a free database of commercially available compounds. They reported the use of various machine learning techniques like Kohonen maps, random forest (RFs) and classification trees to distinguish between metabolites and non-metabolites. Cherkasov [24] and coworkers derived 20 binary classifiers and achieved 99% of the accurate separation between drugs, drug-like compounds ("druglikes"), bacterial and human metabolites and antimicrobial compounds, and proposed metabolite-likeness as a potential tool for discovering novel antimicrobials. Recently, Dobson et al. [25] compared different molecular properties among human metabolites, drugs and "predrugs" (precursor drug molecules). They concluded that although metabolites are a distinct class of compounds, metabolites and drugs occupy a significant amount of common property space. They further suggested that metabolite-likeness may be used as a filter for designing drugs which are functionally similar to metabolites and thus have better ADMET properties.

The several excellent studies described above have each compared different datasets, using a variety of chemoinformatics tools and molecular descriptors. Furthermore, some of the studies used datasets that were clustered [25], while others have searched or compared complete (unclustered) datasets [24]. Most importantly, the property space of toxic compounds has not been included in any of these studies, whereas one of the basic tenets of drug development to reduce or eliminate toxicity [26].

The analysis carried out of the drug failures during past few decades have shown that over 90% of the failures are due to high toxicity [27,28]. It is therefore essential that the property space of toxins is explored along with drugs and metabolites to develop filters for toxicity.

Our aim is to compare freely available datasets of metabolites, drugs and toxins, as benchmark datasets, using a range of available molecular descriptors, to identify the property space occupied by these three data types. We also present analysis results from complete datasets, as well as clustered datasets, to determine whether clustering molecules would affect the analysis results. Our results indicate that clustering does not affect property distributions to a significant level and that unclustered datasets can be used in drug discovery pipelines. We also report, for the first time to the best of our knowledge, that current drug molecules are more akin to toxins than to metabolites, in physicochemical property space.

## Results

### Rule of five (Ro5) analysis

The number of molecules adhering to Ro5 was calculated and the results are reported in Table 1. It is surprising to note that although Ro5 was formulated to pick out drugs or drug-like molecules, it actually does well in identifying toxin molecules. Over 90% of the toxin molecules satisfy all Ro5 criteria. On the other hand, metabolites perform worst among the three datasets while drugs do fairly well, as expected due to the predominance of Ro5 over the past decade. It should also be noted that among the four properties compared, the numbers of hydrogen bond donor and acceptor seem to be more robust properties, as over 84% of the molecules in all the datasets satisfy Ro5 requirements.

**Table 1:** Distribution of molecules following Lipinski's rule.

Datasets	Lipinski Properties			
	
	Molecular weight <500 Da	H-bond Donor <=5	H-bond Acceptor <=10	Log P <5
HMDB (Metabolites)	34%	84%	84%	35%
DDB (Drugs)	84%	86%	87%	92%
CPDB (Toxins)	94%	98%	97%	92%

### Examining the molecular properties of three datasets

**Figure 1:**
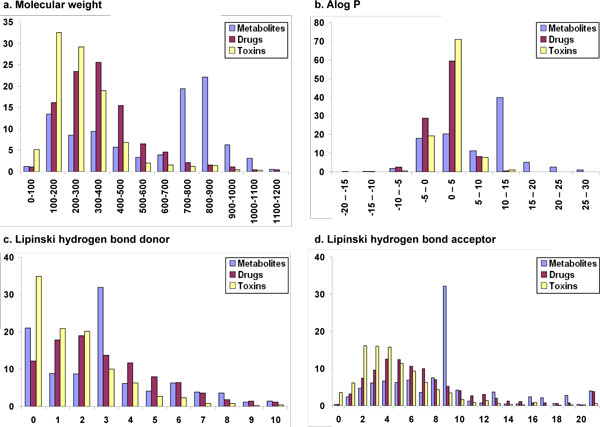
**Comparison of Lipinski properties among human metabolites, drugs and toxins**. Compared properties include a. Molecular weight, b. AlogP, c. Number of Lipinski hydrogen bond donors and d. Number of Lipinski hydrogen bond acceptors.

**Figure 2:**
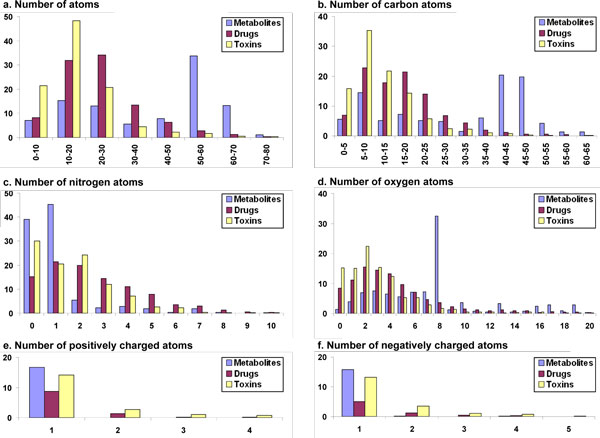
**1D Atomic property differences between human metabolites, drugs and toxins**. Compared properties include a. Number of atoms, b. Number of carbon atoms c. Number of nitrogen atoms d. Number of oxygen atoms e. Number of positively charged atoms f. Number of negatively charged atoms

**Figure 3:**
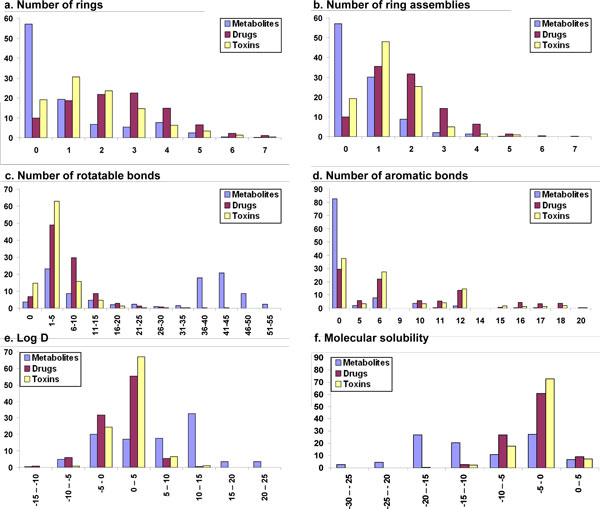
**Other 1D properties compared among human metabolites, drugs and toxins**. Compared properties include a. Number of rings, b. Number of ring assemblies c. Number of rotatable bonds, d. Number of aromatic bonds, e. Log D, f. Molecular solubility.

**Figure 4:**
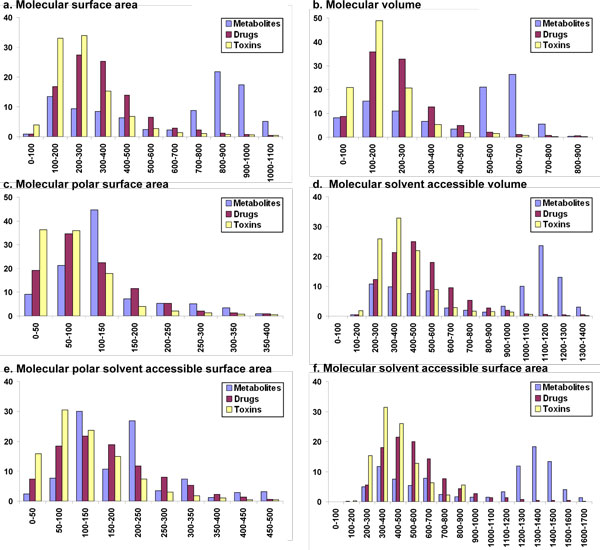
**Comparison of 3D properties among human metabolites, drugs and toxins**. Compared properties include a. Molecular surface area, b. Molecular volume c. Molecular polar surface area, d. Molecular solvent accessible volume, e. Molecular polar solvent accessible surface area, f. Molecular solvent accessible surface area.

**Figure 5:**
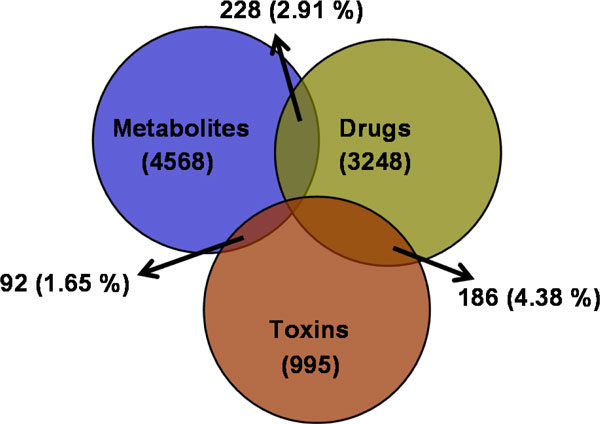
**Venn diagram showing the overlap between the three clustered datasets**.

The distribution of various descriptors (properties) among drugs, human metabolites and toxin molecules are available from Table 2 and Fig. 1, 2, 3, 4, based on the analyses of clustered datasets (details in the Methods section). There is very little overlap in the clustered datasets and so no further reduction in redundant data has been carried out (details in the Methods section and Fig. 5).

**Table 2:** Comparison of molecular properties among the three datasets

	Mean (Median) ± std. dev.		
	
Molecular Property	Metabolites	Drugs	Toxins
***Lipinski properties***			
**Molecular weight**	**621 (701) ± 322**	**355 (309) ± 259**	**275 (239) ± 167**
**Alog P**	**7 (10) ± 7**	**.08 (1) ± 3.5**	**2 (2) ± 2**
**Lipinski HB acceptors**	**9 (9) ± 6**	**7 (6) ± 7**	**5 (4) ± 4**
**Lipinski HB donor**	**3 (3) ± 3**	**3 (3) ± 4**	**2 (1) ± 2**
***1D properties***			
Number of atoms	43 (51) ± 22	24 (21) ± 8	16 (14) ± 11
**Number of carbon atoms**	**34 (41) ± 18**	**16 (14) ± 12**	**12 (10) ± 9**
Number of hydrogen atoms	60 (72) ± 33	23 (19) ± 18	16 (12) ± 12
**Number of nitrogen atoms**	**1 (1) ± 2**	**3 (2) ± 3**	**2 (1) ± 2**
**Number of oxygen atoms**	**8 (8) ± 5**	**5 (4) ± 5**	**3 (2) ± 3**
**Number of rings**	**1 (0) ± 2**	**3 (2) ± 2**	**2 (2) ± 2**
Number of ring assemblies	1 (0) ± 1	2 (2) ± 1	1 (1) ± 1
**Number of rotatable bonds**	**27 (37) ± 20**	**6 (4) ± 7**	**3 (2) ± 4**
**Number of aromatic bonds**	**1 (0) ± 4**	**8 (6) ± 7**	**6 (6) ± 6**
Log D	6 (9) ± 7	0.4 (0.9) ± 4	2 (1.4) ± 2.6
Mol. solubility	-10 (-13) ± 8	-3 (-3) ± 3	-3 (-2) ± 3
***3D properties***			
Mol. SA	651 (788) ± 343	364 (316) ± 252	270 (233) ± 159
Mol. volume	450 (548) ± 244	245 (214) ± 170	179 (153) ± 110
**Mol. polar SA**	**143 (126) ± 94**	**121 (95) ± 117**	**84 (63) ± 76**
Mol. SA volume	866 (1051) ± 420	510 (464) ± 272	401 (366) ± 164
Mol. polar sa SA	216 (195) ± 138	191 (156) ± 173	126 (105) ± 91
Mol. sa SA	1034 (1205) ± 472	578 (523) ± 313	451 (408) ± 187

For each dataset, the mean, median and standard deviation values are provided, with properties ordered as Lipinski properties, 1D properties and 3D properties. HB: hydrogen bond; Mol.: Molecular; sa: solvent accessible; SA: Surface Area.

While there is a multitude of molecular descriptors available for carrying out comparison studies, given the large size of the datasets, we need a set of rapidly computable molecular descriptors, for efficient analysis. Furthermore, to account for 70% of the drugs, Oprea et al. [9] used simple descriptors such as the count of rings and rotatable bonds along with Lipinski descriptors. We have considered a range of 1D and 3D properties for the current analysis. The results are presented as Lipinski (Ro5) properties, 1D properties (non-Ro5 measures) and 3D properties.

#### Lipinski properties

##### Molecular weight

Metabolites follow a bimodal distribution in molecular weight, with the first peak at 100-400 (almost 31% of the dataset) and the second and larger peak at 700-1000, containing 48% of the dataset. On the other hand, the molecular weight of drugs follows a Gaussian distribution with the majority of drugs (82%) under the range of 500. This is in accordance with the Lipinski restriction of weight less than or equal to 500. Despite the Ro5 restriction, 18% of the drug molecules possess a molecular weight in excess of 500. Toxin molecules more or less follow the same pattern as drugs, with the gradual decrease in number of compounds as molecular weight increases from 100 to 500 (Fig. 1a).

From the calculated mean and median values for the molecular weight, it appears that the metabolite data is skewed towards high molecular weight compounds whereas drugs and toxin molecules prefer a lower molecular weight distribution. The statistics of the molecular weight property for the three datasets are available in Table 2.

##### Lipophilicity (Alog P)

Lipid solubility is a direct measure of transport abilities of the compound across biological membranes [29]. Drug molecules should have enough solubility to traverse the membrane but should not be too soluble so as to get trapped in them. Thus, lipophilicity of a compound is of special significance in drug discovery programs. The most commonly used parameter to evaluate lipid solubility is the n-octanol/water partition coefficient (Alog P). Positive values of this partition coefficient correspond to a preference for lipophilic or hydrophobic environment while negative values indicate a preference for lipophobic or hydrophilic environment. It is clear from Table 2 and Fig. 1b, that metabolites in general are more lipophilic than drugs or toxic compounds. Only 17% of the metabolites have negative Alog P values confirming that the majority of the metabolites are lipophilic. On the other hand, 39% of the drugs have Alog P values in negative territory, indicating that two-fifths of the drugs are lipophobic. Like metabolites, only 19% of the toxin molecules have negative Alog P values while the majority of the molecules are in the range 0 to +5 which is much smaller range as compared to metabolites (Fig. 1b).

##### Lipinski hydrogen bond donors

Lipinski hydrogen bond donors (LHBDs) are determined by counting the numbers of OH and NH bonds in each molecule [3]. Approximately 21% of the metabolites, 12% of the drugs and 34% of the toxin molecules do not possess any LHBDs. Almost the same percentage of molecules in the drug (~41%) and toxin (~36%) dataset have one or two LHBDs, respectively, while only 17% of the metabolite dataset has the same number of LHBDs. Only 5% of the toxins, 14% of the drugs and 16% of the metabolites have LHBD greater than five (Fig. 1c).

##### Lipinski hydrogen bond acceptor

Only a fraction of molecules in all the datasets (0.35% of metabolites, 0.40% of drugs and 3.6% of toxins) do not possess Lipinski hydrogen bond acceptors (LHBAs), computed by summing the numbers of nitrogen and oxygen atoms in each molecule [3]. Drugs and toxins follow almost the same distribution with the highest percentage of molecules in the range 2-7 LHBA atoms per molecule. On the other hand, metabolites have a wide spread distribution with an unusually high peak at 9 LHBA (Fig. 1d).

#### 1D properties

##### Total number of Atoms

The distribution of the total number of atoms in metabolites follows a bimodal pattern (Fig. 2a), with the larger peak at 50-70 atoms, containing 47% percent molecules and the smaller peak at 10-30 atoms, containing 28% of molecules. The maximum number of atoms in a metabolite molecule is 124, while the mean value is 43 atoms per molecule. In contrast to human metabolites, the drug dataset follows a bell-shaped curve, skewed towards low numbers of atoms per molecule. Approximately 79% of drugs contain 10-40 atoms per molecule. The average number of atoms per molecule in the drug dataset is 24, while in metabolites, the average is 43. Like drugs, toxin molecules also favor smaller numbers of atoms per molecule, with a mean of 16 and a gradual decrease in the number of compounds as the number of atoms increases per molecule. The majority of the toxin dataset (91%) contains 10-30 atoms per molecule while only 9% of toxin molecules contain 30 or more atoms per molecule. The overall statistics of the three datasets is given in Table 2 and show that metabolites tend to have more atoms than drugs and toxin molecules.

##### Carbon content

Almost half of the molecules in the metabolite dataset have carbon atoms in the range 35-55 while 32% have 5-25 carbon atoms per molecule (Fig. 2b). The carbon atom distribution in metabolites has a mean of 33 atoms and a maximum of 100. On the other hand, drugs have a mean of 18 carbon atoms per molecule, with a maximum of 256 and 76% of drugs have carbon atoms in the range 5-25. Similar to drugs, toxin molecules also seem to prefer fewer carbons. In the toxin dataset, 77% of the molecules have 5-25 carbon atoms, while 16% have five or fewer carbon atoms. Only 7% of the molecules have more than 25 carbon atoms in toxin dataset. The distribution of carbon atoms in the toxin dataset has a mean of 12 and a maximum of 62. From Table 2, we note that metabolites contain more carbon atoms than drugs, which in turn have greater carbon content than toxin molecules

##### Nitrogen content

Approximately 40% of metabolites do not have any nitrogen atom (Fig. 2c), while 45% have only one nitrogen atom and 16% have two or more nitrogen atoms per molecule. In sharp contrast to metabolites, only 15% of drug molecules do not posses nitrogen atoms while 74% of the molecules have nitrogen atoms in the range 1-5. On the other hand, 30% of the toxin molecules are devoid of any nitrogen atom while 66% of toxin molecules contain nitrogen atoms in the range 1-5 and only 3% have six or more nitrogen atoms. From Table 2 and the values presented above, drugs molecules clearly possess the most number of nitrogen atoms, followed by toxin molecules and lastly, metabolites.

##### Oxygen content

For the three datasets, there is a clear reversal of the trend for the oxygen atom distribution compared to the nitrogen atom distribution presented in the previous section. Only 1% of the metabolite molecules do not have an oxygen atom as compared to 8% of drugs and 15% of toxin molecules (Fig. 2d). Furthermore, in metabolite dataset, 73% of the molecules possess oxygen atoms in the range 2-8, compared to 68% of drugs and 65% of the toxins. Metabolites comprise more oxygen atoms than drugs, followed by toxic compounds, with mean values of eight, five and three, respectively (Table 2).

##### Number of negatively and positively charged atoms

The fraction of molecules with a single negatively charged atom in the metabolite dataset (16%) is almost the same as that containing one positively charged atom (17%). While the metabolite dataset contains molecules with more than one negatively charged atom, there are no molecules with more than one positively charged atom. The percentage of negatively charged atoms is smaller in the drug dataset as compared to metabolite dataset (Fig. 2e and Fig. 2f). Only 5% of drug molecules contain a negatively charged atom, with only 2% containing two or more negatively charged atoms, whereas 8% contain one positively charged atom. On the other hand, in the toxin dataset, 13% of the molecules contain one negatively charged atom per molecule and 5% of molecules contain two or more negatively charged atoms, whereas 14% of the molecules in the same dataset contain one positively charged atom per molecule and 4% contain two or higher positively charged atoms. The trend of charged atoms among the three datasets is Metabolites > Toxin molecules > Drugs, with the drug dataset favouring negatively charged atoms over positively charged ones.

##### Number of rings

The distribution of number of rings is shown in Fig. 3a. Although more than 55% of the molecules in the metabolite dataset are acyclic, 19% contain a single ring and 21% contain 2-5 rings. In sharp contrast to metabolites, only 9% of the drugs are acyclic, while almost 60% contains rings, with a three-way distribution (~20% each) between one, two and three rings per molecule. The remaining 23% of drug molecules contains 4-6 rings, the maximum number of rings being 38. In the toxin dataset, 19% of the molecules are acyclic, whereas 68% contain 1-3 rings per molecule. The remaining 10% of toxins contain four or more rings per molecule. Thus, the pattern of ring distribution among the three datasets is Drugs > Toxin molecules > Metabolites.

##### Number of ring assemblies

After removing the non-ring bonds from a molecule the remaining backbone is termed as the ring assembly. As shown in the Fig. 3b, more than half of the molecules (57%) in the metabolite dataset have no ring assembly, while 30% of the molecules have one ring assembly and 13% have two or more ring assemblies per molecule. On the other hand, in the drug dataset, only 10% of the molecules are free of ring assembly, whereas 36% and 32% have one and two ring assemblies, respectively. Furthermore, in the same dataset, 23% molecules contain more than three ring assemblies per molecule. Similar to drugs, most of the toxins possess ring assemblies with only 19% are devoid of any ring assembly, while 45% molecule have a single ring assembly. The percentage of molecules with two ring assemblies in the same dataset is 21% whereas 6% of the molecules have three or more ring assemblies. The pattern of ring assemblies is similar to that obtained for ring distribution in three datasets, being Drugs > Toxin molecules > Metabolites.

##### Number of rotatable bonds

The number of rotatable bonds is a measure of molecular flexibility and is important in determining oral bioavailability of the drugs [30]. Only 4% of the molecules in the human metabolite dataset have no rotatable bonds, whereas 32% have 1-10 rotatable bonds and 47% of the molecules have rotatable bonds in the range 36-50 (Fig. 3c). The mean value for rotatable bond distribution in metabolite dataset is 27, with the maximum number of rotatable bonds in a metabolite molecule being 83 (Table 2). Among the drug molecules, 7% are devoid of rotatable bonds, while 79% of molecules have 1-10 rotatable bonds. Another 12% of the molecules in this dataset have rotatable bonds in the range of 10-20. The mean value for rotatable bonds per molecule in drugs is 6, with a maximum of 170. In contrast to metabolites and drugs, 15% of toxin molecules do not possess any rotatable bonds, while 79% of the molecules contain rotatable bonds in the range of 1-10. The mean value for rotatable bonds in toxin molecules is 3 and the maximum number of rotatable bonds in a toxin dataset is 31. Thus, metabolites are more flexible than drugs and toxins.

##### Number of aromatic bonds

More than 80% of metabolites do not possess any aromatic bond. The remaining metabolites have several aromatic bonds, usually as multiples of five or six. As shown in Fig. 3d, 6% of the molecules have either five or ten aromatic bonds, while 8% of molecules have either six or twelve aromatic bonds. The maximum number of aromatic bonds in metabolites is 36.

In contrast to metabolites, only 29% molecules of drugs have no any aromatic bonds. Of the remaining, 12% and 36%, respectively, of drug molecules have aromatic bonds as multiple of five and six. The maximum number of aromatic bonds in the drug dataset is 62. On the other hand, toxin molecules are predominantly aromatic (61%) with 7% and 42% having aromatic bonds as multiples of five and six, respectively. The maximum number of aromatic bonds in the toxin dataset is 46. The order of aromatic bond distribution is Drugs ≈ Toxin molecules > Human metabolites, with almost half the aromatic bonds in all the three datasets being multiples of five or six.

##### Log D

For solutes that can ionize, the distribution coefficient (D) is the ratio of the sum of the concentrations of all forms of the compound (ionized plus un-ionized) in each of the two solution phases. logD is thus considered a better measure of lipophilicity that Alog P. However, for all three datasets, logD follows the same distribution as Alog P (Fig 3e and Table 2).

##### Molecular solubility

Human metabolites have large range of molecular solubility values for example more than 85% of the molecules in metabolite dataset have molecular solubility in the range of -20 to 0. Drug molecules have a smaller range of molecular solubility as compared to metabolites, with 87% of the molecules in drug dataset having molecular solubility values spanning -10 to 0. Similarly, more than 90% of toxin molecules have a solubility value in the range -10 to 0. Thus, the most preferred and common range of molecular solubility among the three datasets is -10 to 0, which comprises ~85% of the drug and toxin datasets but only 38% of the metabolite dataset (Fig. 3f and Table 2). The molecular solubility of metabolites is more than that of drugs, followed by toxin molecules, which suggests that metabolites tend to dissolve more easily than drugs and toxins *in vivo *(aqueous media).

##### Chirality

Chirality seems to be a distinguishing feature among the three datasets. The majority of the molecules in the metabolite dataset (74%) are chiral. Chirality falls sharply in drugs and toxic compounds to 31% and 14%, respectively.

##### Number of halogen atoms per molecule

As expected, toxin molecules have the highest number of halogen atoms per molecule compared to metabolites and drugs. 31% of molecules in toxin dataset possess a single halogen atom (F, Cl, Br, I) per molecule while in case of drugs close to 18% contain halogen atoms. In sharp contrast, to these two datasets, metabolites have far fewer halogen atoms per molecule. Only 15 out of 4568 molecules, i.e. only 0.3% of the molecules, studied are reported to have any halogen atom. The trend for halogens is Toxin molecules > Drugs >> Metabolites. The statistics provided in Table 3 provides information on the number of halogen containing molecules in each dataset.

**Table 3:** Halogen atom frequency distribution. The number of times different halogens are reported in each of the dataset is listed below.

Database	Fluorine	Chlorine	Bromine	Iodine
Metabolites	15	32	0	27
Drugs	496	477	110	63
Toxins	62	473	38	5

##### Number of Sulphur and Phosphorus atoms per molecule

Only 5% of the molecules in metabolite dataset, 20% of the drugs and almost the same percentage (16%) of toxin molecules contain one or more sulphur atoms in their molecule. For sulphur atoms, the trend is Drugs ≈ Toxin molecules > Metabolites. The trend gets reversed in the case of phosphorus atoms, with 46% of molecules in metabolites, 13% of drug molecules and only 3% of the toxic dataset having one or more phosphorus atoms. So the trend in phosphorus atom distribution is Metabolites >> Drugs > Toxin molecules.

##### Average bond length per molecule

The metabolite molecules form two groups, with 48% having a mean value of 0.82 Å bond length, while another 51% have 0.83 Å. The majority of drug molecules (65%) also have either 0.82 or 0.83 Å bond length. In sharp contrast to the other two datasets, the average bond length for 92% of molecules in toxic dataset is 1.33 Å, while another 7% of the molecules have 1.40 Å as the average bond length. As far as average bond length is concerned, metabolites and drugs have a much shorter average bond length compared to toxin molecules, the trend being Toxin molecules > Metabolites ≈ Drugs.

#### 3D Descriptors: molecular volume and surface area

##### Molecular surface area

Molecular surface area distribution in metabolites is bimodal (Fig. 4a) with the first smaller peak at 100-400 Å^2^, containing 37% of the molecules and the second larger peak at 700-1100 Å^2^, with 53% of the molecules. On the other hand, 83% of drugs molecules have molecular surface area between 100-500 Å^2^. A similar distribution is obtained for toxin molecules with 89% of the compound in the toxin dataset having a molecular surface area in the range 100-500 Å^2 ^and only 4% are in the range 0-100 Å^2^. From these values and the statistics in Table 2, metabolites have greater molecular surface area than drugs and toxin molecules.

##### Molecular volume

The results of molecular volume distribution in three datasets are reflected in the related property of molecular weight distribution. As depicted in Fig. 4b the molecular volume range in metabolites is much wider and in accordance with molecular weight data when compared to the other two datasets. Almost 47% of the molecules have molecular volume in the range of 500-700 Å^3^. The majority of molecular volume distribution of the drug dataset is narrow compared to that of human metabolites with 81% of the molecules are in the range form 100-400 Å^3^, although the tails extend further, with some molecules found to have volumes above 1700 Å^3^. The molecular volume range is even more restricted in toxic compounds with 90% of the molecules in the range 0-300 Å^3 ^with ~49% of these having a molecular volume of 100-200 Å^3^. So, the trend for molecular volume distribution is the same as that observed for molecular weight distribution among the three datasets: Metabolites > Drugs > Toxin molecules.

##### Molecular polar surface area

The polar surface area is defined as the surface area summed over all polar atoms, (usually oxygen and nitrogen), including the attached hydrogen atoms. It is often correlated with drug transport capabilities and is important for penetrating the blood-brain barrier (BBB). As most of the metabolites do not need to be shuttled through barriers like BBB, they can afford to have more polar surface area than drugs and toxins. More than 95% of the metabolites have polar surface area in the range 0-350 Å^2 ^(Fig. 4c) while 92% of polar surface area of drugs is contained within 0-250 Å^2^. The distribution is even narrower for the toxin dataset with 90% of the molecules in the range 0-150 Å^2^.

##### Molecular solvent accessible volume

Molecular solvent accessible volume distribution is similar to the distribution of the molecular volume. In the case of metabolites (Fig. 4d), it also follows a bimodal distribution with a smaller peak of 36% molecules around 200-600 Å^3 ^and a larger peak containing 46% of the molecules around 1000-1300 Å^3^. However, there is no molecule with accessible volume less than or equal to 100 Å^3^. Unlike metabolites, drugs molecules have only one peak covering almost the entire dataset. About 91% of the drug molecules have solvent accessible volume from 200 to 800 Å^3^. Like metabolites there is no molecule with solvent accessible volume less than or equal to 100 in drug dataset. The distribution of solvent accessible volume in toxin molecules is even thinner with 89% of the molecules in the range 200-600 Å^3^. Other 7% are present in the range 600-1000 Å^3^. According to the statistics shown in Table 2 and Fig. 4d, the order of molecular solvent accessible volume is Metabolites > Drugs > Toxin molecules.

##### Molecular polar solvent accessible surface area

Drugs and toxin molecules follow a perfect Gaussian distribution for polar solvent accessible surface area while metabolites follow a bimodal pattern (Fig. 4e). The maximum number of molecules in toxic dataset has molecular polar solvent accessible surface area is in the range 0-200 Å^2 ^while for drugs the range is 0-350 Å^2^. On the other hand maximum numbers of metabolites are covered in between 100-250 Å^2^. The statistics in Table 2 suggests that metabolites tend to have larger molecular polar solvent accessible surface area compared to drugs which in turn are larger than toxins.

##### Molecular solvent accessible surface area

Differences among metabolites, drugs and toxin molecules are readily observable for molecular solvent accessible surface area. Metabolites follow a bimodal distribution whereas drugs and toxins follow a Gaussian distribution (Fig. 4f). Toxin molecules peak at 300-500 Å^2 ^while drugs peak at 400-600 Å^2^. Metabolites, on the other hand, form a lower peak at 300-400 Å^2 ^with a second larger peak at 1200-1500 Å^2^. Form Table 2, metabolites have clearly larger values for molecular solvent accessible surface area than drugs and toxins.

### Functional group analysis

The frequency of functional group occurrence among the three datasets was carried out in this study with the Scitegic Pipeline pilot software (details in the Methods section). The occurrence of specific functional groups of interest to drug design is given in Table 4 and Additional file 1. Aromatic atoms are a prominent feature among drugs and toxins while only a sixth of metabolites have aromatic atoms. The same trend is observed in benzene ring distribution among the datasets. Further, primary and quaternary amines occur more frequently in metabolites than secondary and tertiary amines when compared to drugs and toxin molecules, respectively. Additionally, drugs are found to possess a greater number of amides than metabolites or toxins. Finally, toxic functional groups (like nitro, azo and cyanide) are only found in toxins while they are either absent or very limited in drugs and metabolites.

**Table 4:** Occurrence of functional groups in the three datasets

Functional Group	Metabolite dataset	Drugs dataset	Toxin dataset
Alkyl halide	<0.5%	<0.5%	3.2%
Aromatic atom	17.4%	70.6%	62.3%
Benzene	10.3%	56.0%	53%
Steroid backbone	2.9%	0.6%	<0.5%
HBA Ester	56.3%	13.8%	15.4%
Pyridine	1.2%	6.4%	5.3%
Pyrimidine	3.2%	7.5%	1.9%
Enamine	3.2%	10.31%	3.41%
Primary amine	28%	14.4%	12.0%
Secondary amine	11.4%	64.0%	41.2%
Tertiary amine	44.6%	80.0%	60.0%
Quaternary Amine	15.3%	2.1%	0.5%
Primary amide	1.5%	4.5%	3.9%
Secondary amide	11.4%	31.0%	14.5%
Tertiary amide	2.8%	16.8%	9.2%
Imines	4.1%	14.0%	6.4%
Azo	0%	<0.5%	3.4%
Carbamic acid	<0.5%	3.1%	1.9%
Urea	2.5%	8.0%	6.5%

Those functional groups which can discriminate between the three datasets are presented here. The complete list is in Additional File 1.

### Clustered *vs*. unclustered datasets

**Figure 6:**
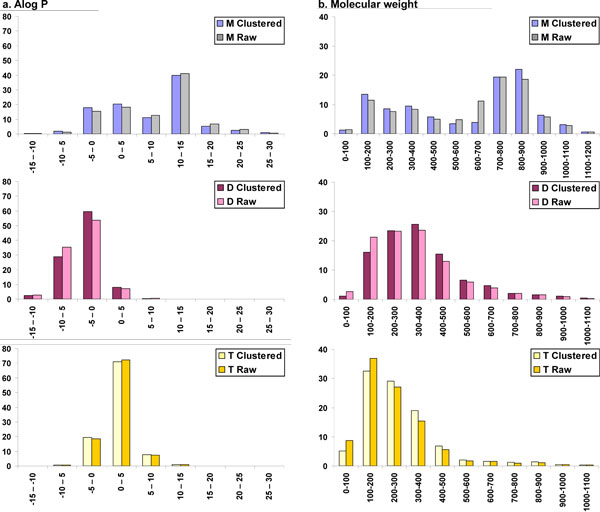
**Comparison of example Lipinski properties for clustered and unclustered (raw) data**. Properties compared are a. Alog P, b. Molecular weight,, for human metabolites (M), drugs (D) and toxin molecules (T).

**Figure 7:**
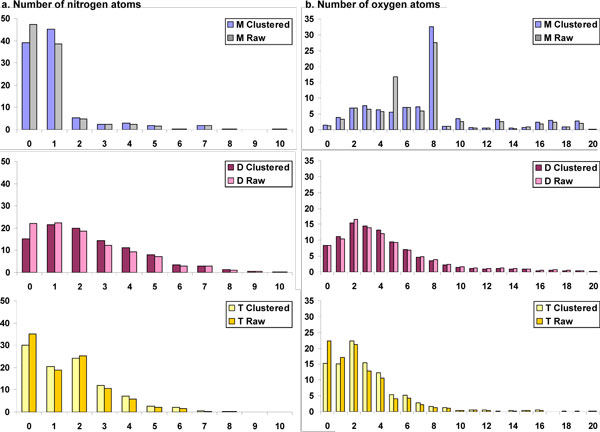
**Comparison of example 1D atomic properties for clustered and unclustered (raw) data**. Properties compared a. Number of nitrogen atoms, b. Number of oxygen atoms, for human metabolites (M), drugs (D) and toxin molecules (T).

**Figure 8:**
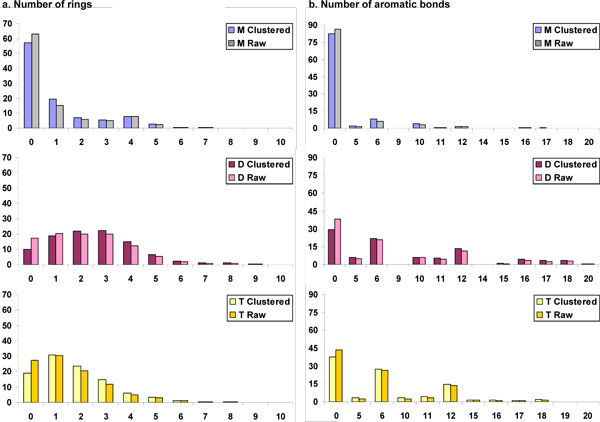
**Comparison of example 1D aromatic properties for clustered and unclustered (raw) data**. Properties compared are a. Number of rings, b. Number of aromatic bonds, for human metabolites (M), drugs (D) and toxin molecules (T).

**Figure 9:**
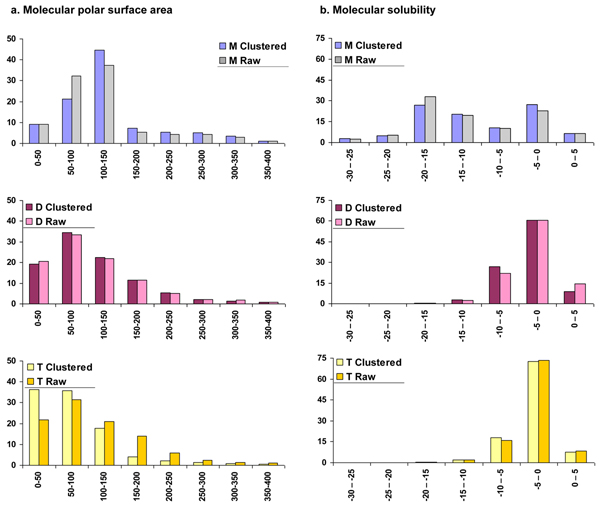
**Comparison of example molecular properties important in drug design, for clustered and unclustered (raw) data**. Properties compared are a. Molecular polar surface area, b. Molecular solubility, for human metabolites (M), drugs (D) and toxin molecules (T).

We have compared all the above property distributions for clustered and unclustered (raw) datasets (data not shown). Correlation coefficients were calculated for all the properties and eight properties which are not significantly correlated are presented here, *viz. *Alog P, molecular weight, the number of oxygen atoms, the number of nitrogen atoms, molecular polar surface area, molecular solubility, the number of rings and the number of aromatic bonds (Figs. 6, 7, 8, 9). Alog P and molecular weight values (Fig. 6) do not deviate significantly with clustering. Nitrogen atom distribution (Fig. 7) for clustered and unclustered molecules also remains fairly similar for all the datasets. The analysis also shows that the number of aromatic bonds (Fig. 8) and the molecular solubility distribution (Fig. 9) are also fairly conserved between clustered and unclustered datasets. We note that, by and large, the two distributions are very similar except in following cases:

#### Number of oxygen atoms

There is an exception at five oxygen atoms per molecule in the unclustered metabolite dataset (Fig. 7b).

#### Number of rings

The number of molecules with zero rings drops for drugs (~8% decrease) and toxins (~9% decrease) whereas metabolites follow a similar distribution in clustered and unclustered dataset comparison (Fig. 8a).

#### Molecular polar surface area

Clustered metabolites show a 10% decrease in molecules with polar surface area in the range 50-100 Å^2 ^while clustered toxins show a 15% increase in the number of molecules with polar surface area between 0 to 50 Å^2^. Drugs, on the other hand, follow a similar distribution for clustered and unclustered datasets (Fig. 9a).

## Conclusion

We have carried out a comprehensive analysis of three publicly available datasets, comprising drug, metabolite and toxin molecules. We have also, for the first time, to the best of our knowledge, compared the distributions of various properties for complete datasets (unclustered data) as well as reduced or clustered datasets. We note that, in the main, the distributions for the two data groups, clustered and unclustered, are very similar, supporting the use of clustered datasets, except in the case of the number of oxygen atoms, the molecular polar surface area and the number of rings. Based on this result, these properties should be treated with caution for lead discovery in drug discovery pipelines with unclustered datasets.

From the analyses of clustered datasets, we find that two-thirds of the human metabolites lie outside the Lipinski universe. On the other hand, over 90% of the toxin molecules abide by Lipinski's rule, implying that since Ro5 does not explicitly take toxicity into account, present-day drugs are consequently similar to toxins than to metabolites.

Results from the analysis of 1D and 3D molecular properties consolidate our finding of drugs and toxins sharing a larger property space, than drugs and metabolites. 1D properties such as the total number of atoms advocate that metabolites are bulky, with more carbon and hydrogen atoms than drug and toxins. This is consistent with the idea that metabolites are produced at the required subcellular location and thus do not need to be transferred from one location to another. In order to design metabolite-like drugs, it would be beneficial to attempt alternative ways for drug delivery, since traditionally, drugs are required to pass through the blood-brain-barrier, which limits the size of drug molecules. Considering the numbers of nitrogen and oxygen atoms, metabolites prefer oxygen over nitrogen containing groups. Above 50% of the metabolites are acyclic while only 9% of the drugs and 19% of the toxin molecules are acyclic. The number of rotatable bonds measuring molecular flexibility and consequently, oral bioavailability, suggests that metabolites are far more flexible than drugs and toxin molecules. Over 70% of the drugs and 62% of toxin molecules are aromatic while only 20% of the metabolites are aromatic. This result is in accordance with the fact that drugs are derived from various sources including NPs which are mostly aromatic in nature. In all the datasets examined, the majority of molecules have negative solubility values, suggesting that a large proportion of these compounds are soluble in aqueous solutions. Chirality falls sharply from metabolites to drugs and toxin molecules while as expected, the number of halogen atoms are found to be higher in toxins than in drugs and metabolites. The average bond length of 90% metabolites and more than 65% of drugs is much smaller than majority of toxins, suggesting multiple bonds in the former datasets. The analysis results from 3D descriptors such as molecular volume and molecular surface area are reflected in the related property of molecular weight and confirm that present day drugs are more like toxins than metabolites.

The analysis also shows that although drugs share a relatively larger property space with toxins than with metabolites, drugs and toxins are two different classes of compounds as reflected in specific physicochemical characteristics. Drugs tend to have higher values for properties such as molecular weight, the number of oxygen atoms, the number of rotatable bonds and molecular polar surface area whereas toxin molecules have considerably higher Alog P and Log D values.

Additionally, empirical rules like the "rule of five" can be refined to increase the coverage of drugs or drug-like molecules that are clearly not close to toxic compounds, because toxicity reduction is one of the key aspects of drug discovery programs. Our results have implications for the analysis of novel compounds in lead discovery pipelines, to uncover novel target molecules.

## Methods

### Preparation of the dataset

Three publicly available databases, relevant to human diseases and their treatment have been used in this study. The human metabolome database [31] contains information on nearly 7000 small molecule metabolites found in human body. Similarly, DrugBank [32] is a comprehensive resource on drugs and drug targets, with detailed chemical, pharmaceutical and medical information on nearly 3000 drug targets and 4800 drugs including >1,350 FDA-approved small drugs and experimental drugs derived from the PDB-Ligand database [33], containing compounds bound to biomolecules. Distributed Structure-Searchable Toxicity (DSSTox) Carcinogenic Potency Database [34] is hosted by the US Environmental Protection Agency's National Center for Computational Toxicology aiming to provide a public data repository on toxicity data. DSSTox contains experimental results and carcinogenicity information for 1547 substances tested against different species.

Preliminary datasets containing 6668 human metabolites from the human metabolome database (as on 23-Dec-2008), 4883 drugs from DrugBank (as on 6-Jan-2009) and 1547 toxin molecules from DSSTox (as on 16-Jan-2009) were extracted.

From these preliminary datasets duplicates and inorganic molecules (individual atoms, metal salts, inorganic oxides, hydroxides, cations and anions) were removed. Any "missing" compounds (either with no or incomplete structure) were also removed. The "cleaned" collections of unique compounds were compiled into analysis datasets containing 6582 metabolites, 4829 drug molecules and 1448 toxin molecules. Finally, clusters were generated from each dataset, using the Cluster "Clara" algorithm embedded in the Scitegic Pipeline Pilot software [35], which is an approximate version of "partitioning around medoids" (pam) method comprising 70% of the entire raw data, similar to that reported in Dobson et al. [25]. Clustering was performed to address the issue of possible overrepresentation of the chemical space, which might bias the analysis results towards these redundant molecules. Representative sets of molecules were produced by employing the extended connectivity fingerprint (ECFP) [36,37] as a molecular descriptor and Euclidean distance was the distance metric selected. ECFP generates an array of structural features by encoding each atom and its molecular environment within a sphere of specified diameter. Cluster centres were selected as the representatives, for clusters containing more than one molecule while singletons were directly used as cluster centres in non-cluster situations. The contents of unclustered and clustered datasets, prepared for analyses are presented in Table 5.

**Table 5:** Clustered and Unclustered datasets

Dataset	Metabolites	Drugs	Toxin molecules
Unclustered	M: 6582	D: 4829	T: 1448
Clustered	CM: 4568	CD: 3248	CT: 995

The overlap among the three clustered datasets (CM, CD and CT) was calculated and it was found that more compounds are common in between drugs and toxin molecules than any other combination. The results are displayed in Figure 5. As the binary overlap is very small (<5%) and the ternary overlap is negligible, the datasets were retained as such, without further size reduction.

### Calculations of the physicochemical properties

The calculation of all the molecular properties was carried out through the Scitegic pipeline pilot [35] and in-house Perl scripts.

Two types of hydrogen bond acceptors and donors were taken into account. Firstly, the Lipinski type donors (sum of OH and NH) and acceptors (sum of N and O atoms) were calculated as defined by Lipinski et al. [3] and then, all available hydrogen bond donors and acceptors were summed up.

The octanol-water partition coefficient was either retained if provided with the data, or was calculated from Scitegic software. The hydrophobicity measure, Alog P, was calculated using the Ghose-Crippen method [38] which takes into account the group contribution to Log P. Another partition coefficient, Log D (the distribution coefficient), which take into account unionized and ionized species, was also calculated. Log D is equal to Log P for unionizable compounds but with ionized species, Log D is considered better than Log P, as it takes ionized species into account, along with unionized forms. A positive value of Log P or Log D suggests a preference to lipophilic surroundings, whereas a negative value indicates preference to lipophobic (or hydrophilic) environment.

Other simple count-based molecular descriptors enumerating aromatic bonds, atoms, carbon atoms, nitrogen atoms, oxygen atoms, hydrogen atoms and rings were also calculated. Beside these, one-dimensional (1D) descriptors calculated include molecular weight and molecular solubility. Three-dimensional (3D) descriptors like molecular volume, molecular surface area, molecular polar surface area and molecular solvent accessible surface area were also computed. The molecular polar surface area is defined as the sum of all the polar atoms (usually oxygen and nitrogen atoms, and the attached hydrogen atoms). This descriptor is often correlated with drug transport capabilities and is important in penetrating the blood-brain barrier.

## Competing interests

The authors declare that they have no competing interests.

## Authors' contributions

VK curated the dataset and conducted the analysis work; SR directed the study and both authors prepared and approved the manuscript.

## Note

Other papers from the meeting have been published as part of *BMC Genomics *Volume 10 Supplement 3, 2009: Eighth International Conference on Bioinformatics (InCoB2009): Computational Biology, available online at http://www.biomedcentral.com/1471-2164/10?issue=S3.

## Supplementary Material

Additional file 1 **Table S1. Occurrence of discriminatory functional groups in the three datasets**. Click here for file
