# Supplementary material for: Physiochemical property space distribution among human metabolites, drugs and toxins
Source: BMC Bioinformatics. 2009 Dec 3;10(Suppl 15):S10. doi: 10.1186/1471-2105-10-S15-S10 (PMC2788350; doi:10.1186/1471-2105-10-S15-S10)
Supplement: Additional file 1 — Table S1. Occurrence of discriminatory functional groups in the three datasets. [file 1471-2105-10-S15-S10-S1.pdf]

Additional File 1

Physiochemical property space distribution among human metabolites, drugs and toxins

Varun Khanna, Shoba Ranganathan

Table S1. Occurrence of discriminatory functional groups in the three datasets.

The functional groups which are most distinguishable among the three datasets are shown in bold.

Functional Group	Metabolite dataset	Drugs dataset	Toxin dataset
Aldehyde	2.1%	1.5%	1.0%
**Alkyl halide**	**<0.5%**	**<0.5%**	**3.2%**
**Aromatic atom**	**17.4%**	**70.6%**	**62.3%**
**Benzene**	**10.3%**	**56.0%**	**53%**
Benzoic Acid	1.0%	3.4%	3.5%
Benzoic acid amide	0.5%	6.3%	1.5%
Indole	2.7%	3.8%	1.5%
Flavone core	0.5%	0.7%	0.7%
Lactam	0.6%	3.6%	4.4%
Prostaglandins	0.7%	<0.5 %	<0.5%
**Steroid backbone**	**2.9%**	**0.6%**	**<0.5%**
**HBA Ester**	**56.3%**	**13.8%**	**15.4%**
SP hybrid atom	<0.5%	2.5%	2.9%
**Pyridine**	**1.2%**	**6.4%**	**5.3%**
**Pyrimidine**	**3.2%**	**7.5%**	**1.9%**
Carboxylic acid	21.0%	24.1%	10.3%
**Enamine**	**3.2%**	**10.31%**	**3.41%**
Enol	<0.5%	1.5%	1.3%
Enol-Ether	5.5%	3.0%	3.3%
Guanadine	2.3%	4.8%	1.8%

**Functional Group**	**Metabolite dataset**	**Drugs dataset**	**Toxin dataset**
**Primary amine**	**28%**	**14.4%**	**12.0%**
**Secondary amine**	**11.4%**	**64.0%**	**41.2%**
**Tertiary amine**	**44.6%**	**80.0%**	**60.0%**
**Quaternary Amine**	**15.3%**	**2.1%**	**0.5%**
**Primary amide**	**1.5%**	**4.5%**	**3.9%**
**Secondary amide**	**11.4%**	**31.0%**	**14.5%**
**Tertiary amide**	**2.8%**	**16.8%**	**9.2%**
**Imines**	**4.1%**	**14.0%**	**6.4%**
Isourea	0%	<0.5%	<0.5%
Nitrate	0%	0%	0%
Nitrite	0%	<0.5%	<0.5%
Nitro	0%	0%	0%
**Nitroso**	**<0.5 %**	**0.6%**	**8.4%**
Oxime-Ether	<0.5%	1.1%	<0.5%
Semicarbazide	0%	0.7%	2.9%
Isocynate	0%	0%	<0.5%
Hydrazine	0%	<0.5%	2.1%
Diazo	0%	<0.5%	<0.5%
Azide	0%	0%	0%
**Azo**	**0%**	**<0.5%**	**3.4%**
**Carbamic acid**	**<0.5%**	**3.1%**	**1.9%**
Carbamic acid ester	<0.5%	2.3%	1.0%
**Urea**	**2.5%**	**8.0%**	**6.5%**
